# Physical and mental health perspectives of first year undergraduate rural university students

**DOI:** 10.1186/1471-2458-13-848

**Published:** 2013-09-15

**Authors:** Rafat Hussain, Michelle Guppy, Suzanne Robertson, Elizabeth Temple

**Affiliations:** 1 School of Rural Medicine, University of New England, Armidale, NSW 2351, Australia; 2 School of Health Sciences, University of Ballarat, PO Box 663, Ballarat, VIC 3353, Australia

**Keywords:** Physical health, Mental health, Well-being, University students, Adolescents, Young adults

## Abstract

**Background:**

University students are often perceived to have a privileged position in society and considered immune to ill-health and disability. There is growing evidence that a sizeable proportion experience poor physical health, and that the prevalence of psychological disorders is higher in university students than their community peers. This study examined the physical and mental health issues for first year Australian rural university students and their perception of access to available health and support services.

**Methods:**

Cross-sectional study design using an online survey form based on the Adolescent Screening Questionnaire modeled on the internationally recognised HEADSS survey tool. The target audience was all first-year undergraduate students enrolled in an on-campus degree program. The response rate was 41% comprising 355 students (244 females, 111 males). Data was analysed using standard statistical techniques including descriptive and inferential statistics; and thematic analysis of the open-ended responses.

**Results:**

The mean age of the respondents was 20.2 years (SD 4.8). The majority of the students lived in on-campus residential college style accommodation, and a third combined part-time paid work with full-time study. Most students reported being in good physical health. However, on average two health conditions were reported over the past six months, with the most common being fatigue (56%), frequent headaches (26%) and allergies (24%). Mental health problems included anxiety (25%), coping difficulties (19.7%) and diagnosed depression (8%). Most respondents reported adequate access to medical doctors and support services for themselves (82%) and friends (78%). However the qualitative comments highlighted concerns about stigma, privacy and anonymity in seeking counselling.

**Conclusions:**

The present study adds to the limited literature of physical and mental health issues as well as barriers to service utilization by rural university students. It provides useful baseline data for the development of customised support programs at rural campuses. Future research using a longitudinal research design and multi-site studies are recommended to facilitate a deeper understanding of health issues affecting rural university students.

## Background

Undergraduate university students comprise a sizeable portion of the younger population and go on to wield a considerable degree of influence in society through the key roles adopted in the future as professionals, senior executives and politicians [1]. The latest OECD report showed that 62% of the young adults in OECD countries were enrolled in tertiary education at universities [2]. The health and well-being of this population group is important, not only due to their potential societal influence, but because many lifestyle related attitudes and habits are formed at this stage and persist across the life span [3-6].

Before describing the background literature, it is useful to highlight some definitional issues associated with the literature concerning the health of younger age groups. Various authors have used ‘late adolescent’ and ‘young adults’ in discussing health issues concerning 18–24 year olds [5,7,8]. We used the term ‘young adults’ in the current paper. Furthermore, we have used the terms ‘health’ and ‘well-being’ to ensure a more holistic approach, encompassing a spectrum of self-reported physical, emotional and mental health issues [9].

Much of the research on the health and well-being of university students, including some of the Australian studies, has focused predominantly on self-reported risky health behaviors such as: smoking [10-12]; drug and alcohol use [13-18]; and unsafe sexual activity [19-23]. In comparison studies on self-rated physical health by university students were less common [1,24-27]. Perhaps the most comprehensive research conducted into the self-rated health of college students was carried out regularly by the American College Health Association. Of the 90,666 students surveyed in 2012, 60.2% rated their health status as excellent or very good, while 31.9% rated it as good [28]. The most common health problems experienced by these students in the past year were allergies (19.7%), sinus infection (17.5%), back pain (12.6%), and strep throat (10.7%). Health conditions which had negatively impacted on their academic performance included: stress (29.0%); sleep difficulties (20.6%); anxiety (20.2%); and upper respiratory tract infections (15.6%). Another recent study found that almost 60% of university students had experienced a health problem in the past month, ranging from allergies and asthma, to severe headaches and insomnia [25].

With regard to mental health, there was considerable more literature and the available evidence suggested that a significant proportion of young adults suffered from psychological ill health. The most up-to-date data from the global burden of disease study showed that mental disorders accounted for four and five out of the ten leading causes of disability-adjusted life-years (DALYs) globally for 20–24 year olds, and 15–19 year olds respectively [7,29]. In Australia, the latest data from the national mental health survey reported the 12-month prevalence of any mental health disorder to be 26% amongst the 16–24 year old group [30]. Similar results have been reported from secondary analysis of other Australian national datasets such as the 2007 Household, Income and Labour Dynamics survey in Australia (HILDA), and the 2007–08 National Health Survey [31]. The mental health of young adults is of concern, not only because of the substantial burden of disease, but because adolescence and early adulthood has been linked to onset for a considerable proportion of mental health disorders diagnosed during adulthood, and due to the persistence of mental illness across the life span [7,29,32-34].

Research studies focused on university students have found that psychological distress is at least as common amongst university students when compared with their age group in the general population. One recent study conducted in the US by Hafen et al., of 78 first-year veterinary science students found 30% of university students rated above the clinical cut-off for depression [35], whilst a large study by Eisenberg et al., of 2785 university students in the US found that that 15.6% of undergraduates tested positive for depression and/or anxiety [36]. Studies in Europe have found similar results. In Hungary in 2008, 19% of students reported considerable psychological distress, with female students scoring significantly worse than their non-student peers in this domain [37]. It is believed that whilst some students commence university with a preexisting mental illness, the stressors associated with university and this stage of life can lead to the manifestation of symptoms in this high risk age group [38,39]. Mental ill-health issues for the university student population can lead to negative outcomes such as: risky health behavior; poor academic performance and attrition; physical illness; antisocial behavior; and suicide [38]. Australian studies involving university students showed a similar pattern [40-43] as found by studies in the US and Europe.

The academic, financial and social challenges associated with university can make this a very stressful time for students [38,44]. There was considerable research on excessive stress leading to burnout in the workplace [45,46], however similar studies into university students’ perceptions and experiences of stress and fatigue, and the associated impact on academic performance and quality of life were relatively limited [24,44,47]. Of the available studies, a large proportion focussed on impact of stress on medical students [47-49]. A study by Vaez et al., in Sweden compared first year university students with their work peers [50]; and a US study by Law found that the level of exhaustion experienced by undergraduate business students was similar or higher than that in conventional high-stress and burnout occupations [51]. As mentioned, much of the burnout research in university students centered on medical students. In the US Dyrbye et al. [48] found 45% of medical students met the criteria for burnout, and further research published in 2008 by Drybye et al. [49] found burnout in 49.6% of medical students. A recent study from South Australia compared rates of psychological distress in undergraduate university students across four distinct disciplinary areas: medicine, psychology, law and mechanical engineering and found slightly higher levels of distress amongst law students compared to medical students [52].

As evident from the information above, most of the research on health and well-being of university students has been conducted in the US and other developed countries. We found only 16 published research studies conducted in Australia since 1995 across a variety of databases including Medline and ProQuest [12,18,20,31,40,42,43,52-61]. An additional two research papers included a systematic review of physical activity across a number of countries [60]; and a recent paper used secondary analysis of national datasets to assess prevalence and correlates of psychological distress in university students compared to their community peers [31]. Only two of the 16 empirical studies were conducted in a rural setting [53,57]. Whilst there was little research around the physical health and well-being of Australian university students, even less is known about their utilisation of available health services. One such study found that although university students were well informed when it came to the services available on campus such as health and counselling facilities, this knowledge did not translate to service usage, with many students having never used the services on offer [43,56].

The aim of the present study was to examine the perceptions of first year undergraduate students studying at a rural university about academic and social stressors and self-rated health. A secondary aim was to examine the accessibility of general practitioners and support services for the students and their peers in a rural university town. Studying as an undergraduate student at a rural campus has its own set of advantages and challenges. The pressures of high-cost accommodation and long-distance commuting of large metropolitan universities are mitigated by being in a smaller rural campus. However, rural communities also have the disadvantage of providing limited anonymity that can be a deterrent from seeking healthcare, particularly in relation to mental health issues.

## Methods

### Sample

The sample frame included all full-time first year students at a public university (the University of New England) located in Armidale, a rural town, in the northern part of the state of New South Wales (NSW). The on-campus students live either in university residential colleges or in town in private accommodation. The student demographics represent the socio-economic and ethnic diversity of the Australian population, where 25-30% of the student population are the offspring of immigrants. Health services include an on-campus medical centre serviced by General Practitioners, which provides services at minimal or no cost through the national insurance (Medicare) scheme. The university also has a free student counselling service.

Ethics approval for the study was obtained from the Human Research Ethics Committee of the University of New England (#HE09/069). An invitation to participate in an online survey was emailed to the sample population by the university’s student services centre in 2009 to all on-campus first-year undergraduate students. This included a summary of the study objectives and a URL address, where potential participants could read the Participant Information Statement and view the online questionnaire before choosing to proceed. Completion of the survey implied consent. As an incentive to participate participants were invited to enter a prize draw to win an iPhone. To ensure integrity of the study in relation to use of a secure and reliable web server, and to maintain anonymity, student services’ staff hosted the survey independently of the research team. The survey was open for ten weeks and two generalised reminders were sent by the student services to all participants. At the end of the survey period, information from completed surveys was made available to the academic researchers in the form of de-identified raw data. A total of 355 students completed the online survey, yielding a response rate of 41%.

### Survey instrument

The survey content for the present study was based on the Adolescent Screening Questionnaire (ASQ), a 52-item validated assessment tool [62]. The ASQ is an Australian instrument developed by the Centre for Adolescent Health at the Royal Children’s Hospital in Melbourne. The instrument was modeled on the internationally recognized HEADSS instrument for screening adolescent health endorsed by the Health Department of the Australian government as part of its national clinical assessment framework for children and young people [63]. HEADSS is an acronym for asking questions about home environment; education/ employment, eating and exercise; activities and peer relation; drug use/ cigarettes/alcohol; sexuality; and suicide/depression/mood. The ASQ was slightly modified as questions were customized to university students only. For example, references to school, vocational college or apprenticeship were removed or substituted with university. Rather than asking whether or not they had considered dropping out of university, they were given an extra option of how much they had thought about dropping out and given five response options ranging from ‘not at all’ to ‘often’, frequently’, and ‘very seriously’. We added an optional “comments” section at the end of the survey form to provide an opportunity for open-ended responses under four sub-headings: on your health; on your well-being, on available services; and other issues affecting university students.

As summarized below, the study instrument consisted of 64 items divided into 11 sections. *Demographic information* included basic questions about the respondent, along with their family structure and accommodation type. *About your education and work* covered how they felt about their studies, how much class had been missed and for what reasons, thoughts of dropping out, and details of paid work and other extracurricular responsibilities. *About your home and family* sought information on how well their family was getting on, whether they could discuss personal concerns with family members, feelings of homesickness, and their perceptions of family communication or contact. *About your friends and activities* covered bullying, participation in group activities, and whether they had a friend they could confide in. *Questions about things you might have done* included recent delinquent behavior, alcohol consumption, cigarette smoking, and drug use. *About your safety* included an additional question on driving whilst under the influence of alcohol or other drugs. *About eating and exercise* measured participation in physical activity, and unhealthy weight loss behaviour. *About your feelings* asked basic mental health screening questions around recent feelings of depression or anxiety on a four-point scale of: ‘never, sometimes, often, and always’, and whether they had ever self-harmed. The eight items for depression and anxiety included feeling anxious in new situations, finding it hard to cope, worry about what other people think, and getting sudden feelings of panic. Items were specifically looking back at the last three months about feeling unhappy and tearful, feeling there was nothing to look forward to, thoughts of dying, and thoughts of self harm. *Questions about sex* explored sexual attraction, age of first sexual activity, safe sex practices, pregnancy, and sexual abuse. *About your health* asked respondents to rate their health on a five-point scale (excellent, very good, satisfactory, poor, not sure). Information was collected on health problems experienced in the past six months, with options including: allergy (skin, food, other); asthma; frequent headaches; fatigue or low energy; skin problems (other than allergy); period problems; and long-term health problems (stomach complaints, muscle or joint pains etc.). Participants were asked whether they had received a diagnosis from a doctor for any illness, about current medication, and the adequacy of access to a General Practitioner (GP) and other support services for themselves, their friends and fellow students.

### Data analysis

Planned analyses included descriptive analyses of demographic, lifestyle and well-being data. In the preliminary analysis frequency distribution of all variables was examined. As this was an exploratory study, post hoc analyses were then conducted to investigate the high prevalence of fatigue reported by the participants. First, a series of one-way ANOVAs was completed to determine if fatigued and non-fatigued groups differed in relation to key demographic, lifestyle and well-being variables. Second, preliminary bivariate Pearson’s correlations were utilised to identify variables for inclusion in a multiple regression analysis, which aimed to determine the combined explanatory value of these variables in relation to the variance in fatigue reported by participants. Finally, as the multiple regression results suggested the presence of a mediated relationship between the predictor variables and fatigue, a model was postulated and tested via structural equation modelling (SEM) and Sobel tests. IBM SPSS Statistics version 20.0 was used for the descriptive, correlational and ANOVA analyses, IBM SPSS AMOS version 20.0 was used for the SEM, and Sobel tests were completed with Preacher and Leonardelli’s Sobel Test Calculator (see: http://quantpsy.org/sobel/sobel.htm). Open-ended responses under the four categories of: your health; well-being; health & support services; and other university services were analysed using thematic analysis [64]. Some verbatim quotes are included in the paper to illustrate particular themes.

## Results

### Participant profile

The survey respondents consisted of 244 (69%) females and 111 (31%) males. The mean age was 20.2 years (*SD* = 4.77). The gender differences are in line with the wider university undergraduate population. Nearly three-quarters of the sample (73%) lived on campus in catered or self-catered accommodation, whilst 16% lived independently or in shared accommodation in town, and 10% lived with their family. Most participants (66%) did not carry out any paid work on a weekly basis. Of the 121 participants who reported paid work, 55% worked less than 10 hours per week, 38% worked between 10–20 hours each week, and 7% worked for 21 to 30 hours per week (see Table 1). A small proportion (12%) of participants had other responsibilities, which were predominantly caring or voluntary work commitments. In relation to coping with academic pressures, 40% of participants had considered dropping out of university during the previous three months. Of these students, 75% had thought about it from time to time, 16% had considered this quite frequently and sometimes quite seriously, while 9% had considered dropping out often and very seriously.

**Table 1:** Socio-demographic profile of study respondents

**Socio-demographics**	**Male**	**Female**	**Total**
	**n (%)**	**n (%)**	**n (%)**
Age (Mean & SD)	Mean 20.7 SD = 6.00	Mean 20.0 SD = 4.10	Mean 20.2 SD = 4.77
Accommodation	78 (70.2)	182 (74.6)	260 (73.2)
On-campus	32 (28.8)	58 (23.7)	90 (25.3)
Private	1 (0.9)	4 (1.6)	5 (1.4)
Other			
Employment			
Don’t work	73 (65.7)	161 (65.9)	234 (65.9)
< 10 hours per week	24 (21.6)	42 (17.2)	66 (18.5)
10–20 hours per week	11 (9.9)	35 (14.3)	46 (12.9)
>20 hours per week	3 (2.7)	6 (2.4)	9 (2.5)

An overwhelming majority (80.8%) of students were non-smokers. Whilst nearly 85% reported consuming alcohol, only a small proportion (10.7%) reported drinking three or more times per week (Table 2). A separate question was asked about frequency of binge drinking in the past month. A third of the sample reported no binge drinking, whilst 15.5% reported 3–4 times in the last month and 10.7% reported frequent binge drinking (5 or more times in the past month). There was a demonstrable gender difference in frequency of binge drinking, 27.9% of the male students compared to 12.3% of the female students (see Table 2). In relation to eating patterns, gender difference was marked with 46.2% of female students (vs. 14.4% of male students) indicating that they had used skipping meals as a strategy to lose weight. A small proportion of female students (10.7%) had skipped meals often/always compared to zero percent of male students (Table 2).

**Table 2:** Distribution of smoking, alcohol and eating behaviours

**Behaviour**	**Male**	**Female**	**Total**
	**n (%)**	**n (%)**	**n (%)**
Smoking			
No	90 (81.1)	197 (80.7)	287 (80.8)
Yes	21 (18.9)	47 (19.2)	68 (19.1)
Alcohol (past month)			
Don’t drink	14 (12.6)	37 (15.1)	51 (14.3)
1–2 times / month	30 (27.0)	109 (44.6)	139 (39.1)
1–2 times per week	43 (38.7)	84 (34.4)	127 (35.7)
>2 times per week	24 (21.6)	14 (5.7)	38 (10.7)
Binge drinking (past month)			
Never (includes don’t drink & never binge)	35 (31.5)	87 (35.6)	122 (34.3)
1–2 times	29 (26.1)	86 (35.2)	115 (32.3)
3–4 times	16 (14.4)	39 (15.9)	55 (15.4)
5 or more times	31 (27.9)	30 (12.3)	61 (17.1)
Skipped meals (past month) for weight loss			
Never	95 (85.5)	129 (52.8)	224 (63.1)
Sometimes	16 (14.4)	91 (37.3)	107 (30.1)
Often	0 (0.0)	17 (6.9)	17 (4.7)
Always	0 (0.0)	7 (2.8)	7 (1.9)

Note: Some columns do not total to 100% due to missing data.

### Self-rated health

Participants rated their health as being excellent (12%), very good (44%), satisfactory (37%), or poor (7%). 80% of participants reported experiencing some health problems over the past six months. An average of 2 (SD = 1.54) health conditions were reported, the most common of which were: fatigue or low energy (56%); frequent headaches (26%); and allergies (24%) (see Table 3). A quarter of the participants had received a diagnosis of a specific illness from a doctor, the most common of which was asthma (13%), followed by anaemia (11%), respiratory infection (9%), and glandular fever (9%).

**Table 3:** Prevalence of self-reported health conditions

**Condition**	**Female n (%)**	**Male n (%)**	**Total n (%)**
Allergy	74 (30.3)	14 (12.6)	88 (24.8)
Asthma	46 (18.9)	13 (11.7)	59 (16.6)
Frequent headaches	75 (30.7)	18 (16.2)	93 (26.2)
Fatigue or low energy	147 (60.2)	53 (47.8)	200 (56.3)
Skin problems	50 (20.5)	16 (14.4)	66 (18.6)
Period problems	67 (27.5)	-	-
Long-term health problems	50 (20.5)	13 (11.7)	63 (17.7)
Other problems	35 (14.3)	20 (18.0)	55 (15.5)
Total	544 (223.0)	147 (132.4)	691 (194.6)

Note: Totals and percentage totals are more than 100% due to multiple responses.

Additional comments made in the open-ended comments section at the end of the questionnaire about self-rated health were mixed. Whilst many commented that their health was “good” or “okay”, many students had experienced frequent episodes of ill-health since commencing university. Poor health was generally attributed to a variety of factors such as: unhealthy food available on-campus in residential colleges; excessive stress from study workloads; juggling study and work commitments; virus transmission due to living in close proximity to others; lack of exercise; constant tiredness and fatigue; and excessive alcohol consumption. A few quotes are provided to illustrate the issues.

“*I think the main issue is the [academic] workload and the social aspect… of expectations of peers. University is a very stressful environment that is hard to maintain a happy medium in.”*

“Being at university has seen my health decline…increase in alcohol and unhealthy food binges during late night study… however, being at college has also encouraged me to exercise as I always have a friend to run or walk with.”

Questions concerning mental health used a four-point response scale (never, sometimes, often, always). Over a quarter of the respondents (26.2%) reported feeling often or always anxious in a new situation, 19.8% often or always found it hard to cope with worries, and 13% reported often or always experiencing sudden feelings of panic. Participants were also asked specifically about their emotional and psychological feelings in the past three months. 21.3% reported often or always feeling unhappy or tearful, nearly 9% often or always felt they had nothing to look forward to, and a small proportion reported often or always feeling so bad that they had thoughts of dying (4.2%) or harming themselves (3.7%) (see Table 4). The majority of participants (85%) had never harmed themselves, however 17% of the females and 11% of the males had tried to harm themselves at some stage during their life. Participants were asked whether they had ever been diagnosed by a medical practitioner as having anxiety or depression. Slightly over 8% of the participants indicated that they were currently, or in the past, diagnosed as having anxiety or depression.

**Table 4:** Frequency of psychological distress

**Feeling**	**Never (%)**	**Sometimes (%)**	**Often (%)**	**Always (%)**
Anxious in new situation	17.5	55.8	20.0	5.9
Hard to cope with worries	24.5	55.2	14.9	4.8
Worry about what others think	22.0	49.3	19.7	8.2
Sudden feelings of panic	47.6	38.6	10.1	2.8
Felt unhappy or tearful	18.6	59.7	17.5	3.7
Felt nothing to look forward to	71.8	19.2	7.0	1.7
Thoughts about dying	83.1	12.4	2.5	1.7
Thoughts about self-harming	84.8	11.3	2.0	1.7

Note: Percentage totals are less than 100% due to missing responses.

The eight mental health items were also combined (via summation) to make a single variable, psychological distress (scoring range 8–32; Cronbach’s α = .87). Female participants (*M* = 14.5, *SD* = 4.36; range: 8–32) scored significantly higher on this variable than male participants (*M* = 12.5, *SD* = 3.22; range: 8–29): *F* (1, 348) = 18.79, *p* < .001, *η*^2^ = .051. Additional comments were also provided in the open-ended category under well-being. The majority of respondents reporting “good” or “great” well-being *“very good, enjoying college, university and all that it has to offer”*. Negative comments indicated that for some students, well-being was being compromised by stressors such as: alcohol consumption; university workloads; relationship difficulties; and missing loved ones.

“*University has… a way of sucking everything out of you and giving you nothing in return. I was generally a happier person until I started undertaking my studies.”*

“My well-being is great at the moment but there are times when I felt down after being dumped by my boyfriend and approaching the end of term and being homesick after 2.5 months away from family.”

Further assessment of quantitative data was undertaken through further bivariate and multivariate analysis. With over half of participants experiencing fatigue or low energy, this variable was examined in greater detail. A series of one-way ANOVAs indicated that fatigue was significantly associated with a number of academic behaviours. Participants reporting fatigue missed more classes (*F* [1, 354] = 15.20, *p* < .001, *η*^2^ = .041); considered dropping out more seriously (*F* [1, 354] = 17.55, *p* < .001, *η*^2^ = .047); and enjoyed their studies less (*F* [1, 353] = 5.05, *p* = .001, *η*^2^ = .030) than those who had not experienced fatigue or low energy. Fatigued and non-fatigued participants differed on a number of health behaviours. People experiencing fatigue over the past six months drank alcohol more frequently (*F* [1, 354] = 6.56, *p* = .003, *η*^2^ = .025); were more likely to regret alcohol related situations (*F* [1, 300] = 7.30, *p* = .007, *η*^2^ = .024); and engaged in more weight loss behaviours (*F* [1, 353] = 8.23, *p* = .004, *η*^2^ = .023); than their non-fatigued peers. Fatigued participants also experienced higher levels of psychological distress (*F* [1, 348] = 22.49, *p* < .001, *η*^2^ = .061); were more likely to have a history of self-harm (*F* [1, 353] = 7.52, *p* = .006, *η*^2^ = .021); and felt unsafe in the previous three months more frequently (*F* [1, 354] = 8.29, *p* = .004, *η*^2^ = .040); than non-fatigued participants.

To understand the factors contributing to fatigue, preliminary correlation analyses were completed. A number of variables were found to be significantly associated with fatigue. However, after consideration of effect sizes, only three variables (with *r* > .200) were considered appropriate for inclusion in the subsequent multiple regression analysis. As the predictor variables used for the multiple regressions in our study (i.e., psychological distress, self-rated health status, and number of health problems experienced in the previous six months) are continuous and without established/validated cut-points for categorisation into binary outcomes (exposed and non-exposed groups) logistic regression analysis was not considered appropriate. The three variables included in the multiple regression analysis include: psychological distress, *r* (347) = .247, *p* < .001; self-rated health status, *r* (351) = .326, *p* < .001; number of health problems experienced in the previous six months, *r* (353) = .588, *p* < .001. While it can be assumed that there will be an association between self-ratings of health status and number of health problems experienced in the previous six months, interestingly the correlation between these variables indicated less shared variance than perhaps would be expected (*r* [351] = .427, *p* < .001). Therefore, both variables were included in the multiple regression analysis with psychological distress. The three variables explained 34% of the variance in fatigue, however only the total number of health problems accounted for a significant proportion of the variance in fatigue scores (see Table 5).

**Table 5:** Multiple regression model for fatigue

**Predictors**	***B***	***SE B***	***β***	***t***	***p***	***sr***^***2***^
Number of health problems	0.17	0.02	0.53	10.58	<.001	.214
Frequency of alcohol use	<0.01	0.01	0.01	0.22	.829	<.001
Self-rated health	0.06	0.03	0.09	1.87	.062	.006

*Note.* Fit for model *R*^2^ = .34, Adjusted *R*^2^ = .34, *F*(3, 343) = 59.85, *p* < .001. The squared semi-partial (*sr*^2^) correlation is derived from the *Part* correlation in SPSS. Predictors were entered simultaneously.

**Figure 1:**
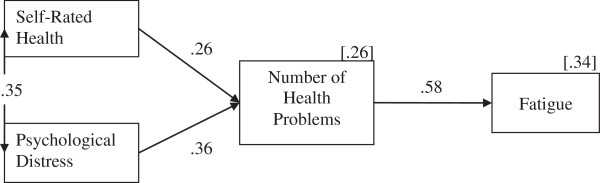
**Model of cross-sectional fatigue predictors and mediator; all relationships are statistically significant (*p*** **< .001).** Fit χ^2^(2) = 4.42, *p* = .110, *CFI* = .992, *TLI* = .975, *RMSEA* = .059 (.000, .135). Values in square brackets are the percentage variance explained.

To gain a more detailed understanding of the relationships between the variables, structural equation modelling (SEM) was employed to test a mediation model, where number of health problems mediates both self-reported health–fatigue and psychological distress and– fatigue relationships (see Figure 1). As can be seen in Table 6, the SEM analyses indicated that there was a good fit between the model and the data. Sobel tests revealed that number of health problems fully mediated the relationship between self-reported health status and fatigue (*z* = 7.08, *p* < .001) and fully mediated the relationship between psychological distress and fatigue (*z* = 6.61, *p* < .001).

**Table 6:** Goodness-of-fit indices for sem model for fatigue

**χ**^**2**^	***df***	**χ**^**2**^**/*df***	***RMSEA***	***CI***_**90c **_**for *RMSEA***		***CFI***	***TLI***
				***LB***	***UB***		
4.42	2	2.21	.059	.000	.135	.992	.975

*Note. RMSEA* root mean square error of approximation, *LB* lower bound, *UB* upper bound, *CFI* comparative fit index, and *TLI* Tucker-Lewis Index.

These findings indicate that there was a positive association between the psychological distress experienced by participants and their self-ratings of poor health. Further, those participants rating their health more poorly and experiencing higher levels of psychological distress reported experiencing a greater number of health problems in the previous 6 months, which in turn, was positively associated with the fatigue experienced by participants. Moreover, there was no direct relationship between self-rated physical health and fatigue or between psychological distress and fatigue.

### Access to medical and support services

As explained in the Introduction section, a secondary aim of the study was to assess access to, and satisfaction with, health services by students studying in a rural town which has limited health services compared to larger regional and metropolitan towns. Information was sought on adequacy of access to General Practitioners [GP] and other support services such as counsellors, for not only the participants, but also their friends and fellow students. The majority of participants (82%) felt they had adequate access to GP and other support services, including counselling. Similarly, 78% reported that there was adequate access to these services for their friends and fellow students. Additional comments provided on services available were varied. Many students found services to be “really good” or “pretty useful”, and in particular were very satisfied with the level of support provided by the residential colleges. The majority of respondents who provided additional comments however were dissatisfied with lack of timely doctor appointments. This was particularly seen as an issue when a doctor’s certificate was required for missing a class, or if applying for a time extension for assignments and exams. With regard to student counselling services, nearly 23% of the sample mentioned a number of issues. The stigma associated with having a mental health problem or being perceived by peers as not being able to cope with academic and social pressures in turn led to mixed emotions of guilt, embarrassment and mistrust, which prevented students from accessing the on-campus counselling services.

“I feel as though help is available, I just need more time to seek it out. Although I know many people would be afraid to seek help, maybe due to embarrassment or shame.”

“I don’t like seeing counsellors because the one at my last high school was condescending. Also I don’t want anyone to know I am seeing a counsellor (if I was to) as I don’t want anyone to know that I am not coping well.”

*“I have used support services to deal with minor issues like sleep deprivation, stress, transition to university and college life. I found the counsellors easy to talk to and well equipped with resources*.*”*

## Discussion

The findings of the present study are in agreement with comparable research on the self-rated health status of university students, with over half the current sample rating their health status as very good or excellent [37,50,65]. Despite the high self-reported health status however, 80% of participants had experienced at least one health condition over the previous 6 months and many reported a substantial decline in their health since commencing university. Health conditions reported were also similar to those found in other research, with a high incidence of allergies, asthma, respiratory infection and persistent or serious headaches [25,65].

In accordance with research into the mental health of university students and the equivalent age group in the general population, it was found that 8% of the students had received a diagnosis of depression or anxiety, and the incidence of psychological distress was elevated. These results are of concern due to both the short-term and potential long-term consequences of mental ill-health. In the short-term, poor mental well-being impairs quality of life, and can result in poor health, social, and educational outcomes including attrition and drop-out [7,29,38]. In the long-term, mental illness with an onset between the ages of 10 years and 24 years is known to persist throughout the life span [32,33].

Fatigue in university students has been reported by a number of studies [54,57,66,67]. Excessive fatigue is often an outcome of the various challenges of university life, which impact both on physical and psychological health, such as meeting academic requirements and standards, weakening of family ties, new and tenuous social support networks and lifestyle and recreational activities pursued by young adults [38,44]. In our study, fatigue proved to be a very common health condition experienced by over half of the participants, and it was shown to be negatively associated with academic conduct in terms of missing classes and considering dropping out of university. Fatigue was found to be to be associated with a number of risky health behaviours particularly around alcohol consumption and unhealthy weight loss behaviours. It is possible that these findings reflected two types of fatigue within the student population; sleep deprivation-related fatigue (assuming that alcohol use was associated with less sleep), and illness-related fatigue. The latter type of fatigue appeared to be more prevalent in the study sample, with the regression and SEM analyses suggesting that the number of health problems experienced in the previous six months was the best predictor of fatigue. However, one of the limitations of the study was not exploring the use of energy drinks, often containing large amounts of caffeine as well as alcohol mixed energy drinks. Recent studies show increasing consumption of energy drinks including mixed energy drinks by university students in the US [68,69] Canada [70] and Italy [71]. A recent Australian study, using focus groups with students, found a similar pattern of popularity of energy drinks and mixed energy drinks [55]. Disturbed sleep pattern, irregular waking hours, and fatigue have been reported as a consequence of increasing use of energy drinks [72].

In relation to barriers to seeking healthcare, previous studies show that accessibility of GP and other support services for the students surveyed and their friends and peers is varied [73]. Barriers to seeking healthcare seem be similar across various studies internationally and nationally and include concerns about privacy, stigma and difficulties with emotional openness [74-77]. In a recent study published in 2012, Stallman [56] reviewed university counselling services in Australia and New Zealand and found a low uptake of university counselling services. In our study of students at a rural university campus, we found that despite limited range of free services available to students outside the university-setting, the most commonly reported barrier to using university services for mental health issues was “perceived stigma” and lack of privacy. Given the findings of this research in terms of the physical and mental health issues experienced by this participant group, it follows that issues and barriers in service accessibility and delivery need further examination. A future study examining the utilisation of customized online resources versus in-person consultation would be of value in increasing the uptake of counselling services.

Before concluding, the limitations of this study need to be considered when interpreting the findings. The present study was a cross-sectional survey and no longitudinal data was available to assess variations in risk behaviors and self-rated health. It is plausible that university students feel more settled after the first year of study as they develop better coping strategies to balance academic and leisure pursuits and consolidate new social networks. However, Australian studies which include university undergraduate students across various years across the degree program, do not report a major improvement in more senior years of the study program compared to first year and also compared to their age peers in the community [31,52]. Although we did not find poor self-rated health in rural university students compared to studies focusing on metropolitan students, there could be limitations in generalizing the findings to other rural campus settings. A longitudinal multi-site rural study would be of value to explore some of the issues in greater detail.

## Conclusions

The findings of the current study have added to the literature on the health status of young people and in particular, rural university students. With the high rates of physical and mental health conditions being found amongst the university student population, the implications in terms of burden of disease are far-reaching. Further, as this population subgroup are at a stage of their life where they are forming health-related behaviors and belief systems, unhealthy habits developed can persist throughout life. This research provides valuable baseline data to develop health promotion programs targeted at university students studying at rural campuses. Online support services and health education programs have considerable potential in improving strategies for self-care and resilience through development of personal skills during this formative stage of young adulthood [78,79]. Further, universities and other higher education institutions are ideally situated to provide avenues for health promotion programs aimed at improving health literacy, behaviour and attitudes and preventing the onset of physical or mental ill-health which can exact such a toll both for the individual and society [3-5,44,47,73]. The authors recommend that more comprehensive longitudinal mult-site research be conducted into the health and well-being of rural university students, and that targeted health promotion programs are developed accordingly.

## Competing interests

The authors declare that they have no competing interests.

## Authors’ contributions

RH, MG and SR designed the study. All authors contributed to the various stages of project conceptualisation, data collection and analysis. All authors contributed to manuscript development and revisions. All authors read and approved the final manuscript.

## Pre-publication history

The pre-publication history for this paper can be accessed here:

http://www.biomedcentral.com/1471-2458/13/848/prepub
